# Electrodeposition of amorphous WO_3_ on SnO_2_–TiO_2_ inverse opal nano-framework for highly transparent, effective and stable electrochromic smart window†

**DOI:** 10.1039/c9ra03084k

**Published:** 2019-05-29

**Authors:** Tam Duy Nguyen, Loo Pin Yeo, Daniel Mandler, Shlomo Magdassi, Alfred Iing Yoong Tok

**Affiliations:** School of Materials Science and Engineering, Nanyang Technological University 50 Nanyang Avenue Singapore 639798 tamnguyen@ntu.edu.sg MIYTok@ntu.edu; Singapore-HUJ Alliance for Research and Enterprise, NEW-CREATE Phase II, Campus for Research Excellence and Technological Enterprise (CREATE) Singapore 138602; Institute of Chemistry, The Hebrew University of Jerusalem Jerusalem 9190401 Israel

## Abstract

In recent years, there has been significant advancement in smart window technologies due to their effectiveness in reducing energy consumption of indoor lighting and air-conditioning in buildings. Electrochromic (EC) materials, in particular, have been widely studied as they provide a simple method for tuning or modulating visible light and infrared (IR) transmittance. In this work, a novel hybrid, multi-layered SnO_2_–TiO_2_–WO_3_ inverse opal (IO) nanostructure has been fabricated *via* dip-coating and electrodeposition process. This hybrid nanostructure allows an electrochromic smart window for effective near infrared (NIR) modulation, with high visible transparency and durable EC cycling stability. The visible transparency of as-fabricated hybrid multi-layered SnO_2_–TiO_2_–WO_3_ IO was measured to be in the range of 67.2–88.0% in the bleached state and 67.0–74.4% in the colored state, respectively. Furthermore, the hybrid nanostructure is also able to modulate up to 63.6% NIR radiation at the wavelength of 1200 nm and maintain approximately 82.6% of its NIR blockage capability after 750 reversible cycles. The hybrid multi-layered SnO_2_–TiO_2_–WO_3_ IO nanostructure in this study can potentially be an effective and stable EC material for advanced smart window technology.

## Abstract 2

Highly transparent multilayered SnO_2_–TiO_2_–WO_3_ inverse opal nanostructure for enhancing effectivity and stability of near-infrared modulation in advanced electrochromic smart window.

## Graphical abstract

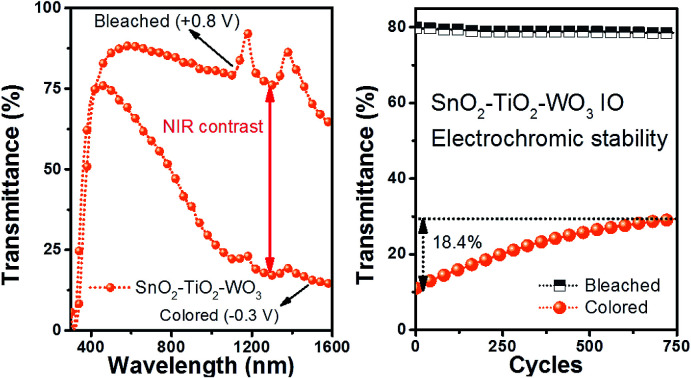

## Introduction

Electrochromic (EC) materials are defined by their ability to change their optical properties reversibly through the application of an electrical current or voltage.^1,2^ Many promising applications that have been proposed, *e.g.* smart glass, electrochromic mirrors, and electrochromic display devices, rely on the phenomenon of electrochromism. EC smart window technology, which is able to electrically modulate the transmittance of solar radiation, is one of the most widely investigated methods to assist in the reduction of energy consumption in indoor lighting and air-conditioning.^3^ This is especially relevant now as more high-rise buildings, are placing enormous strains on energy demands for indoor cooling and heating. Amongst the various EC materials, transition metal oxide WO_3_ is one of the earliest studied materials and still possesses the best EC performance to date.^1^ Upon the electrochemical insertion/extraction of small cations such as H^+^ or Li^+^, WO_3_ exhibits corresponding reversible changes in optical property (colored/bleached) (eqn (1), M = H, Li,…):^4–8^

1 [WO_3_ + M^+^ + e^−^]_bleached_ ↔ [MWO_3_]_colored_

Various material structures and designs have been investigated to improve the overall EC performance of WO_3_, such as nanoparticles,^9–15^ thin films,^16–26^ nanorods^27–29^*etc.* The crystal structure of WO_3_ is also another factor affecting the performance, with many studies reporting that amorphous WO_3_ has better EC efficiency than its crystalline form due to higher ion storage capacity at the same applied voltage.^30–34^ Recently, a modification technique based on three-dimensional inverse opal (IO) structures has been widely used to improve the overall performance of WO_3_ EC material, owing to an enhancement in light absorption with its continuous and periodic structure. In addition, its large specific surface area also increases the active area and improves charge transfer kinetics during redox reactions.^35–41^

Since the high visible transparency is one of the fundamental properties of glass window, it is necessary to shift the radiation modulation to the IR (or NIR) range. On the other hand, the IR (or NIR) modulation is also found to be more effective for the heat management in the field of smart window, as nearly 50% of solar energy comes from IR radiation. However, many studies on WO_3_ EC materials have achieved a large IR (or NIR) modulation, but have failed to get high visible transparency especially in the colored state. For example, Zhou *et al.* reported Ag/WO_3_ nanowires which enable approximately 59% NIR modulation at 1100 nm, and approximately 57.1% transparency at 500 nm.^26^ The bilayer WO_3_ IO structure synthesized by Li *et al.* showed 57.6% NIR modulation at 1100 nm, but only 27.3% transparency at 500 nm.^40^ The hybrid TiO_2_–WO_3_ IO structure synthesized by Ling *et al.* also achieved about 62.5% NIR modulation at 1100 nm, but only 22.7% transparency at 500 nm.^41^ The second issue that the WO_3_-based smart window system needs to overcome is the low device lifespan. Long-term EC performance of WO_3_ materials is found to be suffered from degradation due to the accumulation of trapped ions in the host structure.^42–47^ To improve the stability, many modification methods have been reported, including tuning of the fabrication process,^48^ optimizing periodicity and degree of crystallinity,^49^ and varying of the film thickness.^50^ Wen *et al.* reported that ion-trapping-induced degradation, which is commonly believed to be irreversible, can be successfully eliminated by constant current-driven de-trapping.^51^ However, doping or compositing with metal cations or metal oxides, *e.g.* Nb,^52^ Mo,^53^ and most commonly with Ti,^41,54–60^ are the most effective methods to improve EC cycle stability of WO_3_. Overall, it is still a challenge to develop an optimal material structure and design that can satisfy all requested features of a modern smart window technology, *e.g.* high visible transparency, effective NIR modulation and long-term EC stability.

In this study, we aim to achieve all such features in a WO_3_-based nanostructure using double-layered SnO_2_–TiO_2_ IO framework to produce a multi-layered structure. The polystyrene (PS) opal was used as the initial template to fabricate the SnO_2_ and SnO_2_–TiO_2_ IO framework, after which the electrodeposition method was employed to obtain the hybrid multi-layered SnO_2_–TiO_2_–WO_3_ IO nanostructures. The morphology, crystal structure and surface chemistry of the as-fabricated multi-layered SnO_2_–TiO_2_–WO_3_ IO nanostructures were characterized by Field Emission Scanning Electron Microscopy (FESEM), X-ray diffraction (XRD), Scanning Transmission Electron Microscopy (STEM) coupled with energy-dispersive X-ray spectroscopy (EDX), and X-ray photoelectron spectrometer (XPS), respectively. The optical properties and NIR modulation performance of hybrid multi-layered SnO_2_–TiO_2_–WO_3_ IO nanostructures were examined by Ultraviolet-Visible-NIR (UV-Vis-NIR) spectroscopy under constant potential supply. It was found that the addition of an ultra-thin TiO_2_ layer on SnO_2_ IO framework could effectively improve the long-term EC cycling stability of SnO_2_–TiO_2_–WO_3_ IO nanostructure, while maintaining the effective NIR modulation and high visible transparency in both the colored and bleached states. This study introduces a novel EC material structure for stable and effective NIR-active smart window applications.

## Experimental

### Synthesis of hybrid multi-layered SnO_2_–TiO_2_–WO_3_ inverse opal

Fig. S1† illustrates the fabrication procedure of hybrid multi-layered SnO_2_–TiO_2_–WO_3_ IO nanostructure. Mono-dispersed polystyrene (PS) spheres (Thermo Fisher Scientific) were diluted in DI water and sonicated for 30 min. Clean FTO glasses (1.5 × 2.5 cm^2^) were immersed in the PS dispersion horizontally at 60 °C for 3 days to achieve a self-assembled PS opal template on the FTO glass substrate. In this work, two initial PS opal sizes were used in the fabrication process (392 and 520 nm).

The SnO_2_ IO was obtained by dip-coating the PS opal template in an aqueous SnO_2_ precursor.^61^ The precursor solution was prepared by mixing 15 mL of ethylene glycol (EG) (Sigma-Aldrich) and 3 mmol of SnCl_4_·5H_2_O (Sigma-Aldrich) in a glass beaker under continuous stirring, then heated up to 120 °C for 1 h. After cooling of the mixture, 35 mL of ethanol (Sigma-Aldrich) was added into the solution to reduce the viscosity. The PS opal template was dipped twice into the resultant solution under vacuum for 3 min each time and left to dry at 60 °C for 20 min. Vacuum dipping, in this case, assisted the infiltration of the precursor into the PS opal structure. This was followed by calcination at 500 °C for 2 h (ramping rate 1 °C min^−1^) to obtain the SnO_2_ IO. Similarly, a thin TiO_2_ layer was deposited onto the SnO_2_ IO surface by dipping into the TiO_2_ precursor. The standard recipe of the TiO_2_ precursor solution for the dip-coating process was prepared by mixing 1 mL of titanium tetra-butoxide (Sigma-Aldrich), 0.4 mL of hydrochloric acid (37%, Sigma-Aldrich), 16 mL of ethanol (Sigma-Aldrich), and 10 mL of DI water, respectively. The SnO_2_ IO framework was vacuum-dipped into the precursor solution once for 45 seconds and then dried at 60 °C for 20 min. The dip-coated samples were heated at 500 °C for 2 hours at a heating rate of 1 °C min^−1^ to obtain the double-layered SnO_2_–TiO_2_ IO framework.

Finally, WO_3_ electrodeposition was carried out on the SnO_2_–TiO_2_ IO structure to fabricate the hybrid multi-layered SnO_2_–TiO_2_–WO_3_ IO nanostructure. The WO_3_ precursor solution was prepared by mixing 1.03 g of Na_2_WO_4_·2H_2_O (Sigma-Aldrich) and 0.65 mL of H_2_O_2_ (Sigma-Aldrich) in 250 mL of DI water. After that, 2 mL of HClO_4_ (Sigma-Aldrich) was gradually added into the solution until the pH reached the value of 1.2.^62^ The electrodeposition process was conducted at a constant potential of −0.7 V for 200 seconds. SnO_2_–WO_3_ and TiO_2_–WO_3_ IO nanostructures were also prepared as the references using the same parameters. The obtained samples were rinsed with DI water and dried with nitrogen gas.

### Characterization

The morphologies of the obtained samples were characterized by FESEM (JEOL 7600F). The crystal structure of SnO_2_–TiO_2_–WO_3_ IO was characterized by an X-ray diffractometer (Shimadzu XRD-6000, with the *λ* Cu-Kα = 0.15418 nm). The atomic compositions and core–shell structure were identified by a STEM (JEOL 2100F STEM equipped with INCA EDS detector). The surface chemistry is characterized by a Kratos AXIS Supra X-ray photoelectron spectrometer.

The spectro-electrochemical properties of obtained samples were measured using a Solartron potentiostat (Model 1470E) and a UV-Vis-NIR spectrophotometer (Shimadzu UV 3600). The UV-Vis spectra of all samples were recorded in the wavelength range of 300 to 1600 nm at potentials of −0.3 and +0.8 V. The dynamic optical transmittance was also recorded for the SnO_2_–TiO_2_–WO_3_ IO samples in the wavelength range of interest under repeating square wave potentials oscillating between +0.8 and −0.3 V at a time step of 120 s and 80 s respectively. It was recorded for more than 750 reversible cycles to characterize its long-term stability.

## Results and discussion

### Morphology, crystal structure and surface chemistry

**Fig. 1:**
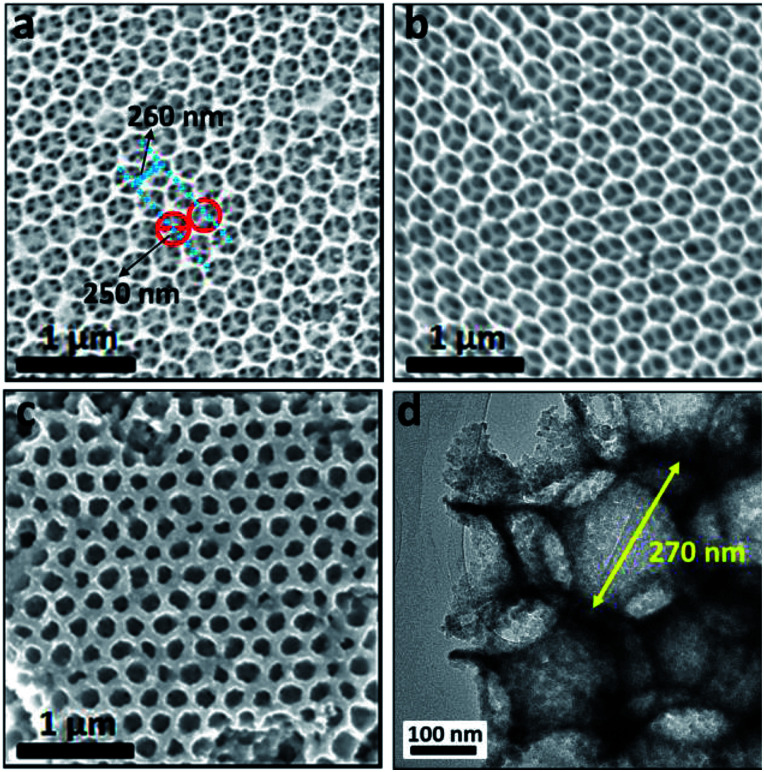
FESEM images of SnO_2_ IO (a), SnO_2_–TiO_2_ IO (b), and hybrid multi-layered SnO_2_–TiO_2_–WO_3_ IO nanostructure (c) with initial pore-size of 392 nm and electro-deposition duration of 200 seconds. All images were acquired under the same magnification. (d) HRTEM image of hybrid multi-layered SnO_2_–TiO_2_–WO_3_ IO nanostructure.

Fig. 1 shows the FESEM images of as-synthesized SnO_2_, SnO_2_–TiO_2_ and hybrid multi-layered SnO_2_–TiO_2_–WO_3_ IO nanostructure with initial PS opal size of 392 nm. The SnO_2_ IO framework (Fig. 1a) is homogenously fabricated after the removal of PS template. The diameter of the SnO_2_ IO pores is approximately 250 nm, which is smaller than that of the initial PS opal diameter (392 nm) due to shrinkage in the SnO_2_ framework following the calcination process at 500 °C. The SnO_2_ IO framework is arranged in a hexagonal array and the openings that connect the adjacent pores can be clearly observed. The distance between two adjacent pores is measured to be approximately 260 nm. With the coating of a thin layer of TiO_2_, there is no significant change in the morphology of the double-layered SnO_2_–TiO_2_ IO framework (Fig. 1b). However, following WO_3_ electrodeposition, a uniformly deposited WO_3_ layer can be clearly observed on the surface of the double-layered SnO_2_–TiO_2_ IO framework (Fig. 1c), forming a hybrid multi-layered SnO_2_–TiO_2_–WO_3_ IO nanostructure. The HRTEM image also confirms the inverse opal structure of as-fabricated SnO_2_–TiO_2_–WO_3_ nanostructures, with the void space of SnO_2_ framework approximated at 270 nm (Fig. 1d). Samples fabricated from 520 nm initial PS opal template have similar morphologies to the 392 nm samples, except with bigger void space dimensions (Fig. S2†).

**Fig. 2:**
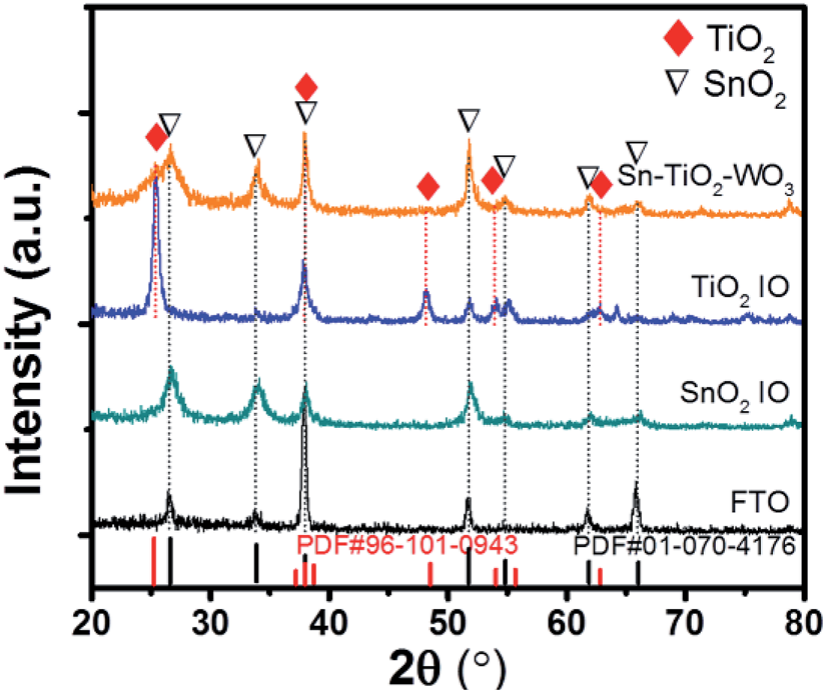
XRD patterns of FTO, SnO_2_ IO, TiO_2_ IO and hybrid multi-layered SnO_2_–TiO_2_–WO_3_ IO nanostructure.

Fig. 2 shows the XRD patterns of FTO substrate, SnO_2_ IO, TiO_2_ IO and hybrid multi-layered SnO_2_–TiO_2_–WO_3_ IO samples. It is observed that both FTO substrate and SnO_2_ IO exhibit characteristic diffraction peaks at 2*θ* = 26.7, 33.8, 38.0, 51.7, 55.0, 61.7, 66.1, 78.7°, which correspond to the planes of (110), (101), (200), (211), (220), (310), (301) and (321) of SnO_2_ (ICDD 01-070-4176), respectively. There is a dissimilarity in the (200) plane peak intensity of both samples, which may be due to the different preferred crystal orientation in SnO_2_ IO structure as compared to the bare FTO glass. The XRD pattern of TiO_2_ IO indicates the formation of anatase TiO_2_, with characteristic diffraction peaks at 2*θ* = 25.4, 37.9, 48.1, 54.0, 62.8°, corresponding to the planes of (101), (004), (200), (105), and (211) of TiO_2_ (ICDD 96-101-0943), respectively. The XRD pattern of multi-layered SnO_2_–TiO_2_–WO_3_ IO shows only the formation of SnO_2_ and TiO_2_ crystal with a lack of distinct WO_3_ peaks, which implies the amorphous nature of the electrodeposited WO_3_ layer.

**Fig. 3:**
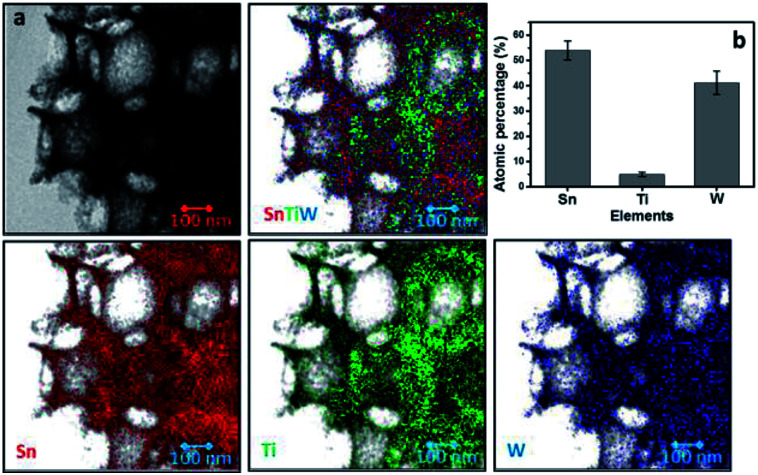
STEM images coupled with EDX mapping of hybrid multi-layered SnO_2_–TiO_2_–WO_3_ IO nanostructure (a) and bulk elemental composition obtained from EDX analysis.

The STEM image coupled with EDX mapping, as shown in Fig. 3a, reveals the multi-layered structure of as-fabricated SnO_2_–TiO_2_–WO_3_ IO. The distribution of SnO_2_, TiO_2_ and WO_3_ layers can be observed in the multi-layered IO nanostructure mapping, which clearly shows that the inner SnO_2_–TiO_2_ framework is surrounded by a layer of amorphous WO_3_. As shown in Fig. 3b, the bulk elemental composition of as-fabricated SnO_2_–TiO_2_–WO_3_ IO is measured to be 53.9, 5.0 and 41.1 at% for Sn, Ti and W, respectively. This indicates that the thickness of the TiO_2_ layer is very thin as compared to the SnO_2_ and WO_3_ layers.

**Fig. 4:**
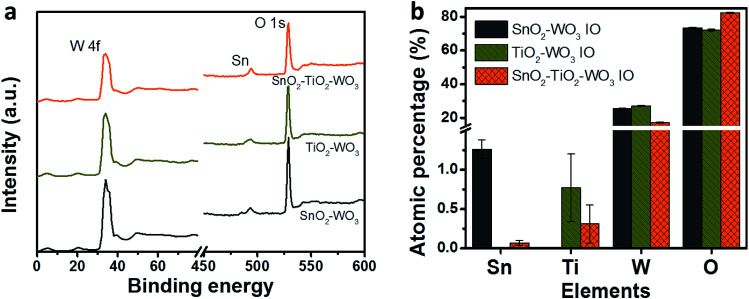
Wide scan XPS spectra (a) and surface elemental composition (b) of SnO_2_–WO_3_, TiO_2_–WO_3_, and hybrid multi-layered SnO_2_–TiO_2_–WO_3_ IO nanostructure.

The surface chemistry of hybrid multi-layered SnO_2_–TiO_2_–WO_3_ IO nanostructure is characterized by XPS measurement. Fig. 4 presents the wide scan XPS spectra and surface elemental compositions of SnO_2_–WO_3_, TiO_2_–WO_3_ and hybrid multi-layered SnO_2_–TiO_2_–WO_3_ IO nanostructures. The wide scan spectra (Fig. 4a) demonstrate the presence of O1s, W4f and Sn3d peaks in all samples, while the peak of Ti2p is negligible due to the low content of TiO_2_ as mentioned in the above EDX bulk analysis. By quantification analysis, the surface elemental composition of SnO_2_–WO_3_ IO is composed of approximately 1.3, 25.4 and 73.3 at% of Sn, W and O, respectively. For TiO_2_–WO_3_ IO, this value is 0.8, 27.2 and 72.0 at% for Ti, W and O, respectively. For hybrid multi-layered SnO_2_–TiO_2_–WO_3_ IO, it is measured to be about 0.1, 0.3, 17.2 and 82.4 at% for Sn, Ti, W and O, respectively. The higher amount of W as compared to Sn and Ti reveals the dominance of WO_3_ layer on the surface of hybrid multi-layered SnO_2_–TiO_2_–WO_3_ IO. The bulk EDX analysis, however, shows that the amount of W (41.1 at%) and Ti (5 at%) is much lower than Sn (53.9 at%) in the hybrid multi-layered SnO_2_–TiO_2_–WO_3_ IO. The results of both analyses correspond well with the expected arrangement in the multi-layered structure of hybrid SnO_2_–TiO_2_–WO_3_ IO.

**Fig. 5:**
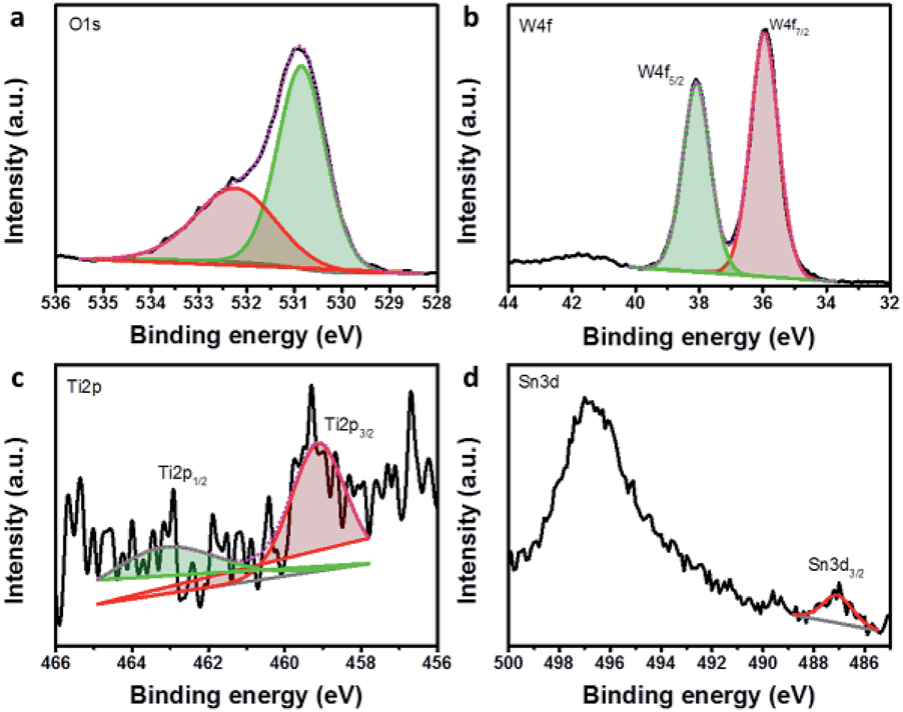
The fine XPS spectra of O1s (a), W4f (b), Ti2p (c), and Sn3d (d) components of hybrid multi-layered SnO_2_–TiO_2_–WO_3_ IO nanostructure.

Fig. 5 presents the fine scan XPS spectra of O1s, W4f, Ti2p and Sn3d components of hybrid multi-layered SnO_2_–TiO_2_–WO_3_ IO. By curve-fitting analysis, the O1s spectrum of hybrid SnO_2_–TiO_2_–WO_3_ IO is located in the metal oxide binding energy range (∼530.8 eV). The W4f spectrum is located at the binding energy of approximately 36.0 eV, confirming the WO_3_ chemical state, with well separated spin–orbit components (*Δ*_metal_ = 2.17 eV). The Ti2p spectrum also confirms the TiO_2_ chemical state (∼459.1 eV). The fine scan XPS spectra of SnO_2_–WO_3_ and TiO_2_–WO_3_ IO nanostructure can also be found in the ESI (Fig. S4).†

### Optical properties and NIR modulation performance

**Fig. 6:**
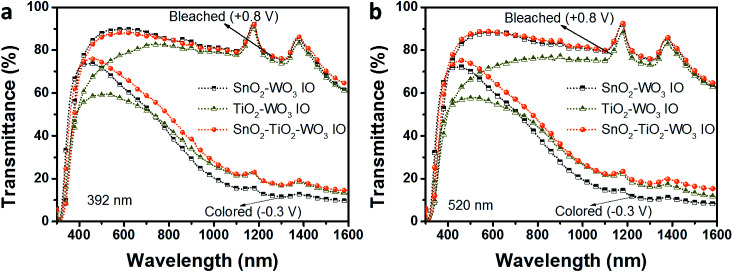
UV-Vis-NIR spectra of SnO_2_–WO_3_, TiO_2_–WO_3_, and hybrid multi-layered SnO_2_–TiO_2_–WO_3_ IO nanostructure at bleached and colored state in 0.1 M LiClO_4_/PC electrolyte. Each UV-Vis-NIR spectrum is obtained from the average of three independent scans.

Since FTO glass allows less than 30% IR transmittance beyond 1600 nm, this work will focus only on the NIR modulation within the range of 700–1600 nm, while the visible transparency is examined between 300–700 nm.^41,61^Fig. 6 shows the UV-Vis spectra of SnO_2_–WO_3_, TiO_2_–WO_3_ and hybrid multi-layered SnO_2_–TiO_2_–WO_3_ IO recorded at constant potentials of +0.8 V and −0.3 V from 300 to 1600 nm in 0.1 M LiClO_4_/PC electrolyte. It has been reported that the WO_3_ IO structure has much higher electrochromic performance than that of WO_3_ thin film.^41,61^ Therefore, in this work, only IO structures obtained from initial PS sizes of 392 (Fig. 6a) and 520 nm (Fig. 6b) are characterized. In the bleached state, all IO samples demonstrate high transmittance in both NIR and visible regions. Upon applying a negative voltage in the colored state (−0.3 V), all samples show a significant reduction in NIR transmittance while maintaining highly transparent in the visible region. The ability to modulate NIR transmittance over a wide range while remaining transparent satisfies the desired optical properties of hybrid multi-layered SnO_2_–TiO_2_–WO_3_ IO for smart window applications. For both tested pore sizes, the visible transparency of hybrid multi-layered SnO_2_–TiO_2_–WO_3_ IO is mostly similar to SnO_2_–WO_3_ IO, and significantly higher than that of TiO_2_–WO_3_ IO.

**Fig. 7:**
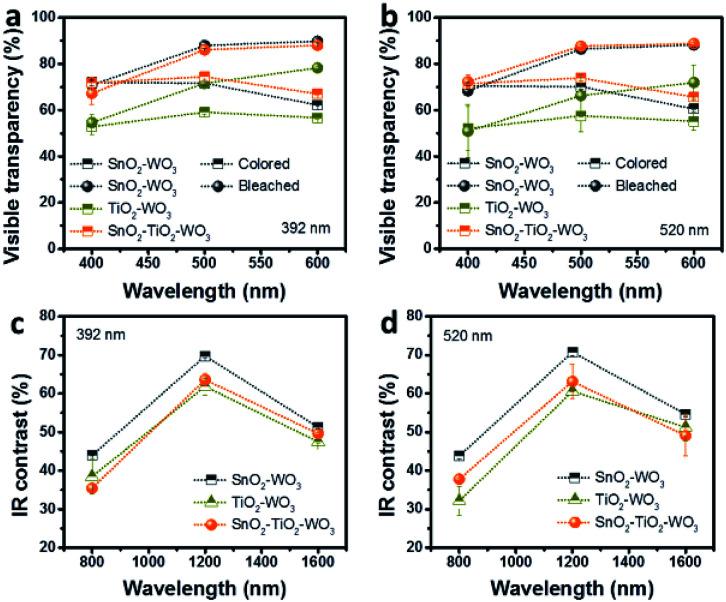
Visible transparency (a and b) and NIR contrast (c and d) between colored and bleached state of SnO_2_–WO_3_, TiO_2_–WO_3_, and hybrid multi-layered SnO_2_–TiO_2_–WO_3_ IO nanostructure.

The detailed electrochromic performance of SnO_2_–WO_3_, TiO_2_–WO_3_ and hybrid multi-layered SnO_2_–TiO_2_–WO_3_ IO nanostructures are presented in the UV-Vis spectra in Fig. 7. Data at several wavelengths were specifically selected for better comparison of the modulation effect: 400, 500, 600 nm (Fig. 7a and b) in the visible region and 800, 1200, 1600 nm (Fig. 7c and d) in the NIR region. In Fig. 7a, 392 nm SnO_2_–WO_3_ IO indicates a visible transparency of 70.8–89.8% during the bleached state and 62.2–70.0% during the colored state. The transparency of 392 nm TiO_2_–WO_3_ IO is lower at 54.6–78.2% during the bleached state and only 52.8–56.7% during the colored state. The hybrid multi-layered SnO_2_–TiO_2_–WO_3_ IO shows a similar transparency as SnO_2_–WO_3_ IO: 67.2–88.0% in the bleached and 67.0–74.4% in the colored states. The same trend of visible transparency was observed for all samples of 520 nm initial PS opal size (Fig. 7b). In comparison with other research, the visible transparency of as-fabricated hybrid multi-layered SnO_2_–TiO_2_–WO_3_ IO nanostructure is found to be much higher, especially in the colored state. Zhou *et al.* reported Ag/WO_3_ nanowires with approximately 57.1% transparency at 500 nm.^26^ The bilayer WO_3_ IO structure synthesized by Li *et al.* showed only 27.3% transparency at 500 nm.^40^ The hybrid TiO_2_–WO_3_ IO structure synthesized by Ling *et al.* also achieved only 22.7% transparency at 500 nm.^41^ This significant improvement in visible light transparency is due to the excellent optical transparency of the SnO_2_ framework as compared to TiO_2_.

The NIR contrast between the bleached and colored states of SnO_2_–WO_3_, TiO_2_–WO_3_ and hybrid multi-layered SnO_2_–TiO_2_–WO_3_ IO nanostructures is presented in Fig. 7c and d. In general, the SnO_2_–WO_3_ IO demonstrates the highest NIR modulation capability, while the hybrid multi-layered SnO_2_–TiO_2_–WO_3_ IO reveals better NIR modulation than the TiO_2_–WO_3_ IO. The 392 nm SnO_2_–WO_3_ IO (Fig. 7c) shows 44.0, 69.7 and 51.2% NIR modulation at 800, 1200 and 1600 nm respectively while TiO_2_–WO_3_ IO shows 38.5, 61.2 and 47.5% NIR modulation at 800, 1200 and 1600 nm, respectively. For the hybrid multi-layered SnO_2_–TiO_2_–WO_3_ IO, the NIR modulation is recorded to be approximately 35.5, 63.6 and 49.6% at 800, 1200 and 1600 nm, respectively. For the samples with 520 nm initial PS opal size (Fig. 7d), the NIR contrast at 800, 1200 and 1600 nm is measured to be 43.8, 70.7 and 54.6% for SnO_2_–WO_3_ IO, 32.1, 60.6 and 51.2% for TiO_2_–WO_3_ IO, about 37.8, 63.1 and 49.1% for hybrid multi-layered SnO_2_–TiO_2_–WO_3_ IO, respectively. The results imply that the NIR modulation performance of SnO_2_–WO_3_ IO structure is not influenced by the presence of an ultra-thin TiO_2_ layer on SnO_2_ surface. In comparison with other reports, as-fabricated hybrid multi-layered SnO_2_–TiO_2_–WO_3_ IO demonstrates comparable NIR modulation performance. The Ag/WO_3_ nanowires reported by Zhou *et al.* showed approximately 59% NIR modulation at 1100 nm;^26^ the bilayer WO_3_ IO structure synthesized by Li *et al.* showed approximately 57.6% NIR modulation.^40^ The hybrid multi-layered SnO_2_–TiO_2_–WO_3_ IO demonstrated 56.9 and 57.6% NIR modulation at 1100 nm for 392 and 520 nm initial PS opal sizes, respectively. Therefore, the hybrid multi-layered SnO_2_–TiO_2_–WO_3_ IO nanostructure developed in this study can provide two promising advantages in the smart windows technology: (1) large thermal radiation modulation and (2) high visible transparency. These advantages will be beneficial for the optimization of in-door lighting and thermal management.

**Fig. 8:**
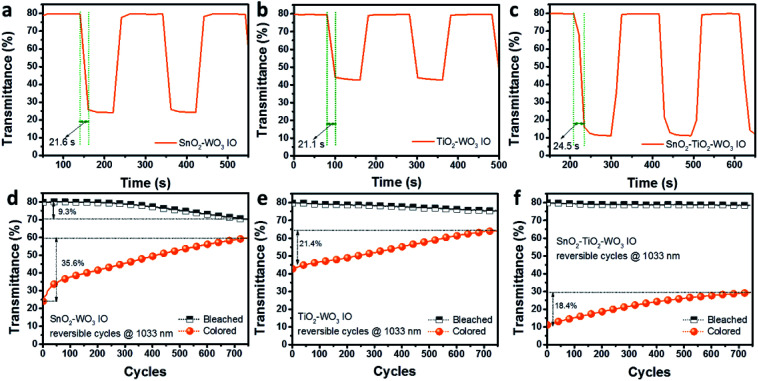
Electro-optical response time between bleached and colored state (a–c) and kinetics stability (d–f) of SnO_2_–WO_3_, TiO_2_–WO_3_, and hybrid multi-layered SnO_2_–TiO_2_–WO_3_ IO nanostructure for over 750 cycles.

The electro-optical response time in switching between the bleached and colored states of SnO_2_–WO_3_, TiO_2_–WO_3_ and hybrid multi-layered SnO_2_–TiO_2_–WO_3_ IO nanostructures is presented in Fig. 8a–c. The switching time is recorded to be approximately 21.6 and 21.1 s for the SnO_2_–WO_3_ and TiO_2_–WO_3_ IO, respectively. The switching time taken for the hybrid multi-layered SnO_2_–TiO_2_–WO_3_ IO is slightly longer, *i.e.* 24.5 s. Fig. 8d–f show the UV-Vis kinetics spectra of SnO_2_–WO_3_, TiO_2_–WO_3_ and hybrid multi-layered SnO_2_–TiO_2_–WO_3_ IO at 1033 nm for 750 reversible cycles. The electrochromic capacity of SnO_2_–WO_3_ IO was found to reduce gradually over 750 cycles for both the bleached and colored states. It is estimated that the electrochromic performance reduces by 9.3 and 35.6% in the bleached and colored states, respectively. The electrochromic cycling stability of TiO_2_–WO_3_ IO shows improvement, with only 4.7 and 21.4% reduction in stability in the bleached and colored states respectively after 750 cycles. The novel hybrid multi-layered SnO_2_–TiO_2_–WO_3_ IO indicates the most significant improvement in EC stability. After 750 cycles, only 1.5 and 19.4% reduction in stability were recorded during the bleached and colored states, respectively. These results suggest that the presence of a thin TiO_2_ layer on SnO_2_ IO framework can significantly improve EC stability, and thus maintain the NIR modulation capability in hybrid multi-layered SnO_2_–TiO_2_–WO_3_ IO nanostructure.

It was reported that lithium accumulates as OLi in the WO_3_ films, but such phenomenon does not occur in WO_3_–TiO_2_ films.^54^ This ion-trapping may be the cause of degradation in the electrochromic performance during bleaching of WO_3_-based EC materials. Consequently, there is an improved EC stability in TiO_2_–WO_3_ structures. In the current study, the results in Fig. 8d–f indicate that the kinetics of ion extraction process (bleached state) is stable for over 750 cycles and only the ion insertion process (colored state) is affected. This implies that there is a reduction in the amount of Li^+^ ions that can be intercalated into the SnO_2_–WO_3_ IO structure after each cycle. This may due to the crystallization of amorphous WO_3_ layer following the reversible cycling test as observed in a previous study.^61^ Arvizu *et al.* also reported that the addition of Ti significantly promoted the amorphous nature of the films and stabilized their electrochemical cycling performance and dynamic range for electrochromism.^56^ This could be the origin for the stable EC performance of as-fabricated hybrid multi-layered SnO_2_–TiO_2_–WO_3_ IO.

## Conclusions

In this work, a novel hybrid multi-layered SnO_2_–TiO_2_–WO_3_ inverse opal (IO) nanostructure is fabricated as an EC material for smart window application *via* electrodeposition of amorphous WO_3_ on a double-layered SnO_2_–TiO_2_ IO framework. The as-fabricated hybrid nanostructure integrates the high visible transparency of SnO_2_, effective near infrared (NIR) modulation of amorphous WO_3_, and durable EC cycling stability of TiO_2_. It is measured that the novel hybrid multi-layered SnO_2_–TiO_2_–WO_3_ IO is able to modulate up to 63.6% NIR radiation at the wavelength of 1200 nm, while still allowing high visible transparency of approximately 67.2–88.0% and 67.0–74.4% in the bleached and colored states, respectively. Furthermore, it can also maintain approximately 82.6% of its NIR blockage capability after 750 reversible cycles. The smart window technology based on this hybrid multi-layered SnO_2_–TiO_2_–WO_3_ IO nanostructure WO_3_ can effectively assist in light and thermal management in buildings, thus reducing energy consumption of indoor lighting and air-conditioning.

## Conflicts of interest

There are no conflicts to declare.

## Supplementary Material

RA-009-C9RA03084K-s001
