# Supplementary material for: Electrodeposition of amorphous WO3 on SnO2–TiO2 inverse opal nano-framework for highly transparent, effective and stable electrochromic smart window
Source: RSC Adv. 2019 May 29;9(29):16730–7. doi: 10.1039/c9ra03084k (PMC9116115; doi:10.1039/c9ra03084k)
Supplement: RA-009-C9RA03084K-s001 [file RA-009-C9RA03084K-s001.pdf]

Electronic Supplementary Material (ESI) for RSC Advances.
This journal is © The Royal Society of Chemistry 2019

Supplementary Information

Electrodeposition of amorphous WO_3 on SnO_2-TiO_2 inverse opal nano-framework for highly transparent, effective and stable electrochromic smart window

Tam Duy Nguyen [a,b,], Loo Pin Yeo [a,b], Daniel Mandler [b,c], Shlomo Magdassi [b,c], Alfred Iing Yoong Tok [a,b,*]*

[a]School of Materials Science and Engineering, Nanyang Technological University, 50 Nanyang Avenue, Singapore 639798

[b]Singapore-HUJ Alliance for Research and Enterprise, NEW-CREATE Phase II, Campus for Research Excellence and Technological Enterprise (CREATE), Singapore 138602

[c]Institute of Chemistry, the Hebrew University of Jerusalem, Jerusalem 9190401, Israel

*E-mail: tamnguyen@ntu.edu.sg / MIYTok@ntu.edu.sg

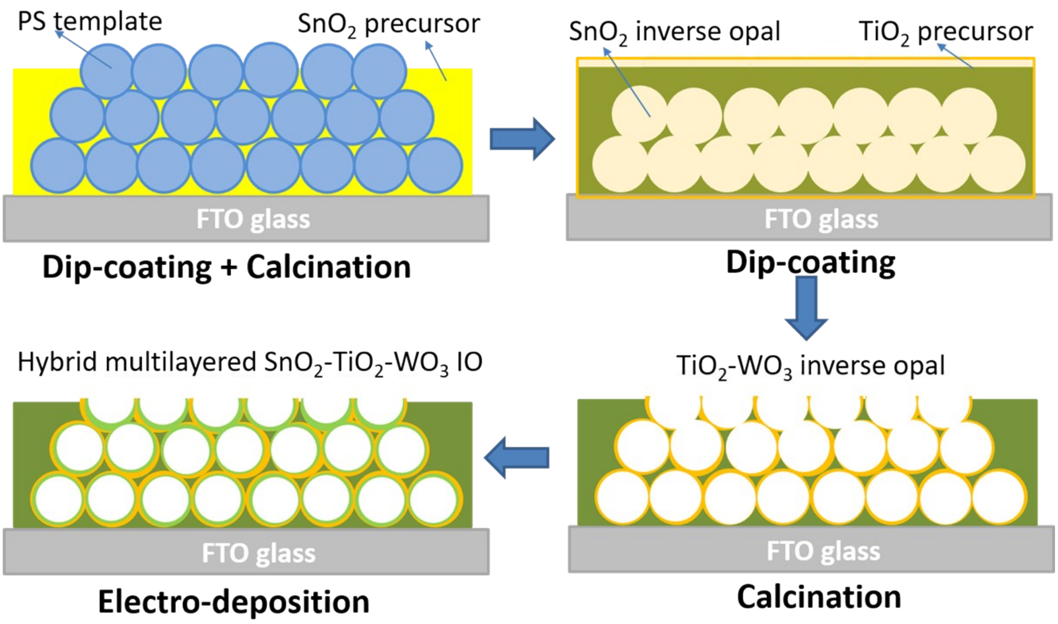

Figure S1: The synthesis procedure of hybrid multilayered SnO_2-TiO_2-WO_3 inverse opal nanostructure.

The hybrid multilayered SnO_2-TiO_2-WO_3 inverse opal nanostructure was fabricated onto a 1.5 x 2.5 cm^2 commercial FTO glass. The as-received FTO glass was pre-treated by a cleaning procedure using methanol, acetone and ethanol, and finally kept in the mixed solution of ethanol and DI water before sample fabrication. The PS spheres were dispersed in DI water solvent with the concentration of 0.02 wt.%. For each fabrication, three pieces of pre-cleaning FTO glass were immersed in 100 mL of PS dispersion solution.

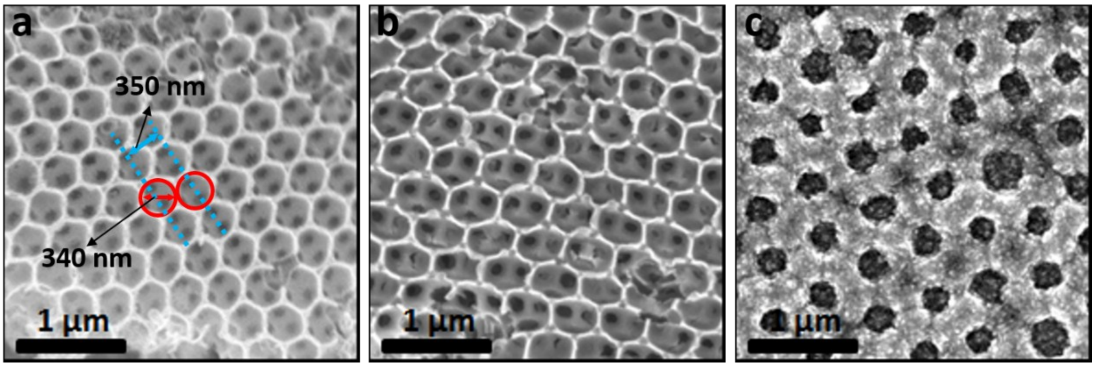

Figure S2: FESEM images of SnO_2 IO (a), SnO_2-TiO_2 (b) and hybrid multilayered SnO_2-TiO_2-WO_3 IO nanostructure (c) with initial pore-size of 520 nm and electro-deposition duration of 200 seconds. All images were acquired under the same magnification.

Figure S2 shows the FESEM images of SnO_2, SnO_2-TiO_2, and hybrid multilayered SnO_2-TiO_2-WO_3 IO nanostructure obtained from the PS solution with initial pore-size of 520 nm. The diameter of the SnO_2 IO pores is about 340 nm, which is smaller than that of the initial PS opal diameter (520 nm). The distance between two adjacent pores is measured to be approximately 350 nm. With the coating of a thin layer of TiO_2, there is no significant change in the morphology of the double-layered SnO_2-TiO_2 IO framework (Figure S2b). However, following WO_3 electrodeposition, a uniformly deposited WO_3 layer can be clearly observed on the surface of the double-layered SnO_2-TiO_2 IO framework (Figures S2c), forming a hybrid multilayered SnO_2-TiO_2-WO_3 IO nanostructure.

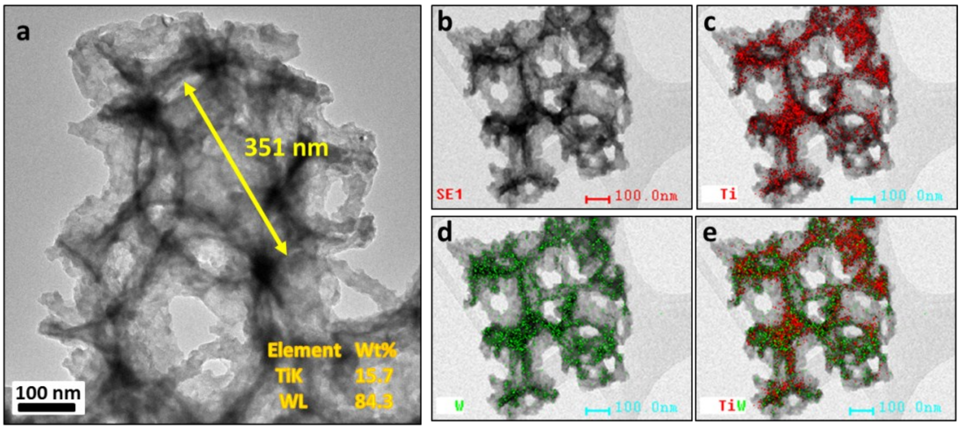

Figure S3: STEM images coupled with EDX mapping of hybrid multilayered SnO_2-TiO_2-WO_3 IO nanostructure obtained from the PS solution with initial pore-size of 392 nm.

Figure S3 indicates the STEM image coupled with EDX mapping of TiO_2-WO_3 IO nanostructure. It is obvious that the inner SnO_2 framework is surrounded by a layer of amorphous WO_3. By EDX analysis, the bulk elemental composition of TiO_2-WO_3 IO is measured to be 15.7 and 84.3 at.% for Ti and W, respectively.

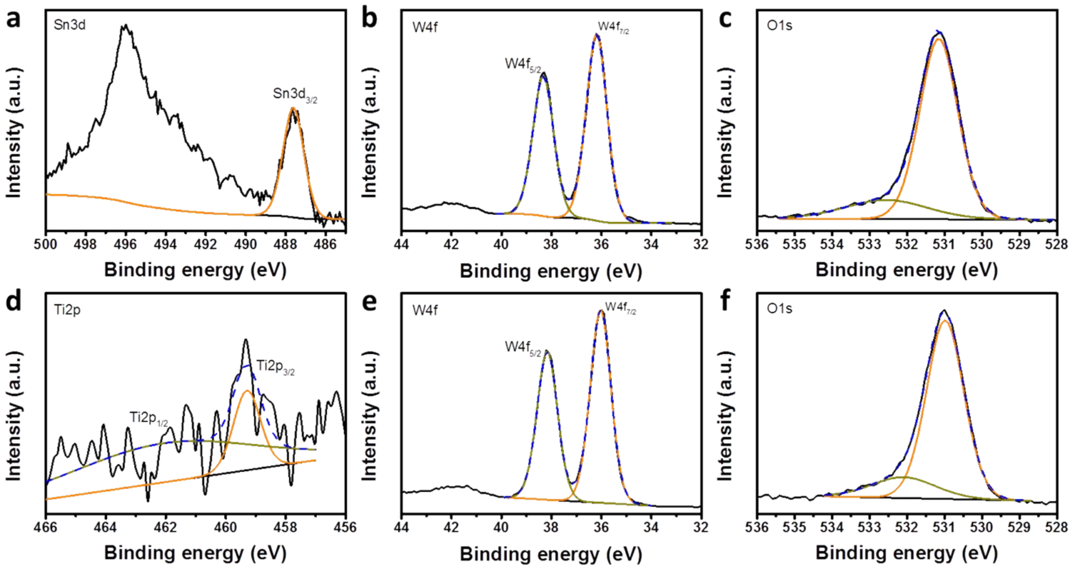

Figure S4: The fine XPS spectra of SnO_2-WO_3 (a-c) and TiO_2-WO_3 (d-f) IO nanostructures.

Figure S4 presents the fine scan XPS spectra of Sn3d, W4f, O1s components for SnO_2-WO_3 and Ti2p, W4f, O1s for TiO_2-WO_3 IO nanostructures. By curve-fitting analysis,

the O1s spectrum of both SnO_2-WO_3 and TiO_2-WO_3 IO is located in the metal oxide binding energy range (~530.8 eV). The W4f spectrum is located at the binding energy of approximately 36.0 eV, confirming the WO_3 chemical state, with well separated spin-orbit components (Δ_{metel}=2.17 eV). The Sn3d and Ti2p spectra also confirms the SnO_2 (~486.6 eV) and TiO_2 chemical state (~459.1 eV), respectively.
